# Corticoefferent pathology distribution in amyotrophic lateral sclerosis: *in vivo* evidence from a meta-analysis of diffusion tensor imaging data

**DOI:** 10.1038/s41598-018-33830-z

**Published:** 2018-10-18

**Authors:** Martin Gorges, Kelly Del Tredici, Jens Dreyhaupt, Heiko Braak, Albert C. Ludolph, Hans-Peter Müller, Jan Kassubek

**Affiliations:** 1 Department of Neurology, University of Ulm, Ulm, Germany; 2 Clinical Neuroanatomy, Department of Neurology, University of Ulm, Ulm, Germany; 3 Institute of Epidemiology and Medical Biometry, University of Ulm, Ulm, Germany

## Abstract

A sequential transaxonal disease spread of amyotrophic lateral sclerosis (ALS)-associated TDP-43 pathology in four stages has been defined by *post-mortem* data, which have been transferred to *in vivo* imaging by diffusion tensor imaging (DTI) studies. Here, we aimed to investigate whether DTI meta-data are consistent with this proposed pattern of progression in ALS. A systematic literature search using the search engines PubMed and Scopus yielded a total of 370 publications. Of these, 57 studies with cross-sectional data and 10 longitudinal studies of human whole-brain analyses of fractional anisotropy (FA) were included in the final data analysis. Statistical meta-analyses on coordinates of significant FA alterations were performed on a grand average alteration data set using a fixed-effect model. A widespread pattern of white matter impairment was identified from cross-sectional meta data (*n* = 2064 ALS patients vs. *n* = 1688 controls) and supported from longitudinal meta data (*n* = 266 ALS patients over 8 months). The results from cross-sectional meta-analyses corresponded to the brain regions and tract systems according to the sequential disease spread of ALS. Structural alterations in ALS patients vs. controls followed a power gradient, i.e., the most frequent alterations were observed along the corticospinal tract (CST, related to ALS stage 1), followed by frequent alterations along the corticorubral/-pontine tract (related to ALS stage 2), together with corticostriatal pathways (related to ALS stage 3), and, finally, alterations in the hippocampal regions adjacent to the proximal portion of the perforant path (related to ALS stage 4). The results from the DTI-based neuroimaging meta-analysis strongly support the model of the corticoefferent axonal disease progression in ALS and provides further *in vivo* evidence for the proposed staging scheme of ALS-associated pathology.

## Introduction

Amyotrophic lateral sclerosis (ALS) is the most frequent adult-onset motor neuron disease and is characterized clinically by a rapidly progressive paresis leading to death within approximately three years^1^. It includes sporadic presentations without known causal pathogenic mutations (95% of all cases) in addition to a heterogeneous hereditary group (5% of all cases, including *SOD1* and *FUS* ALS), the latter of which clinically resemble the sporadic disease but display diverse histopathologies^2^. *Post-mortem* studies of brains from patients with sporadic ALS permitted the definition of a four-stage corticoefferent sequential axonal spread of phosphorylated 43 kDa TAR DNA-binding protein (pTDP-43)^3,4^. This corticoefferent spreading model includes different clinical subtypes, such as bulbar or spinal site-of-onset^5^. ALS patients with a family history, including cases with the *C9orf72* hexanucleotide expansion, showed the same distribution pattern of pTDP-43 pathology as apparently sporadic cases^5^. This *post-mortem* staging model has been transferred *in vivo* to MRI-based concepts by *in silico* models^6^, microstructural data^7,8^, and additional functional connectivity evidence^9^. In particular, *in vivo* evidence has been provided by fiber-tract of interest (TOI)-based diffusion tensor imaging (DTI) data analysis of fractional anisotropy (FA), i.e., a hypothesis-driven approach that revealed sequential involvement of the corresponding white matter tracts in cross-sectional^7^ and longitudinal data^8^.

FA decreases initially in the corticospinal tract (CST, related to stage (1), then in the corticorubral/-pontine tract (related to ALS stage 2), followed by the corticostriatal pathway (related to ALS stage 3), and, finally, the proximal portion of the perforant path (related to ALS stage 4)^10^. The *in vivo* staging categorization with sequential involvement of these specific white matter structures has been consistently demonstrated at a group level^7^ and from longitudinal data at the individual level^8^. Following the concept of meta-analysis, we hypothesized that the analysis of clustered DTI-based studies within the framework of a systematic meta-study in both cross-sectional and longitudinal studies would result in the identical pattern of tract structures that become sequentially involved during the disease course according to the ALS propagation concept. This study aimed to include a large number of DTI-based studies matching the inclusion criteria, such that >2000 ALS patients and >1600 controls are contributing to the final meta- analyses to see if the results of studies with a lower number of ALS participants performed prior to the proposed ALS-staging theory in 2013^3^ are comparable to recently published large-scale multicenter DTI study. We further hypothesized that the resulting alterations from the meta-analysis with respect to their frequency resembled the pattern of sequential involvement of ALS-associated tracts and in pathways associated with the *in-vivo* staging scheme^8^.

## Results

### Study inclusion

**Figure 1:**
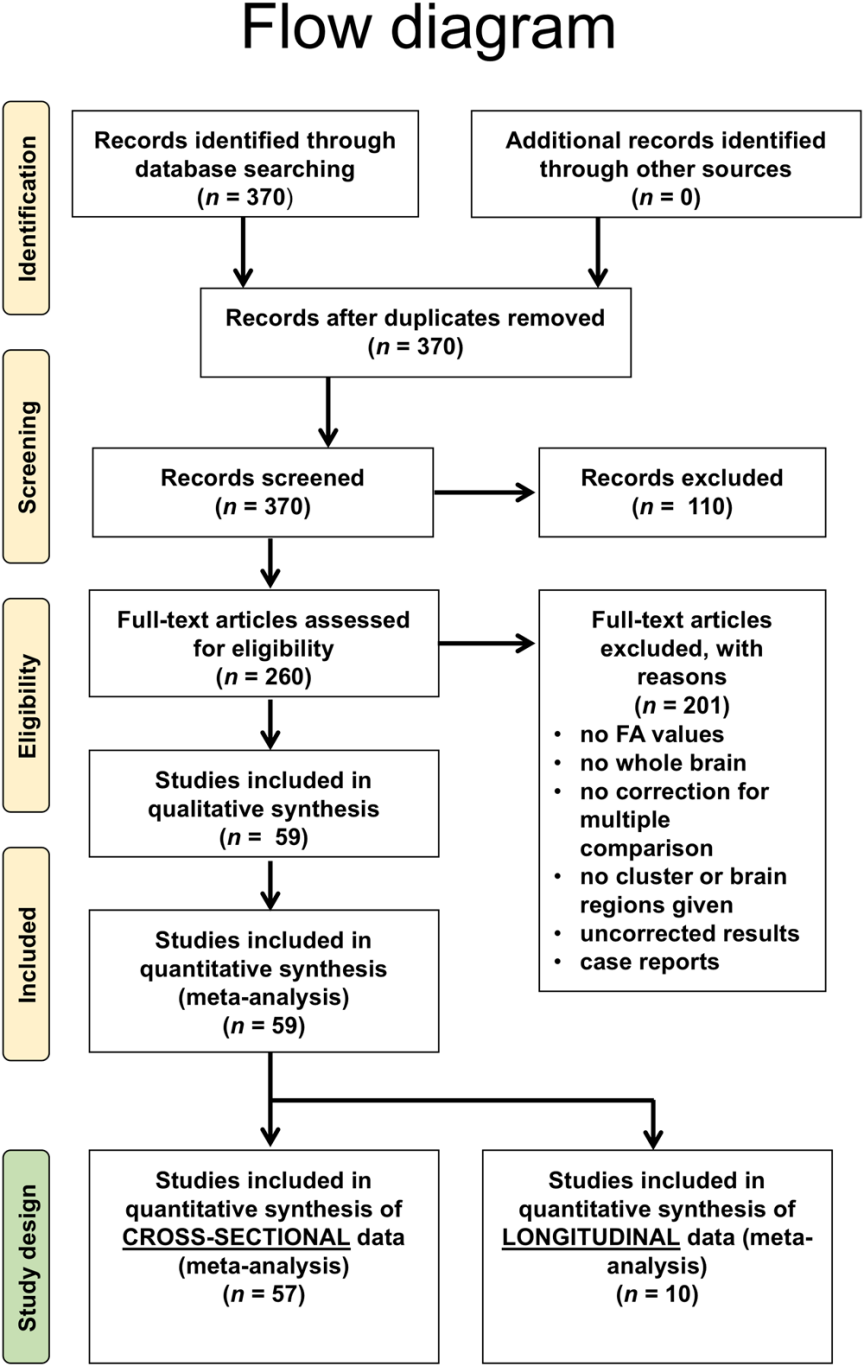
Study selection and corresponding statistics. Flow diagram of the study selection procedure.

The search strategy and study inclusion (Fig. 1) were carried out according to the PRISMA guidelines^11^ and yielded a total of 370 publications. Of these, 59 studies met the inclusion criteria. Fifty-seven studies were subjected to the meta-analyses of cross-sectional data and 10 studies were subjected to the longitudinal meta analyses (Fig. 1). Two studies out of 59 had a longitudinal design without cross-sectional data and, thus, could not be used for the cross-sectional meta analysis, thereby leaving 57 studies for inclusion in the cross-sectional meta study.

In 57 studies with cross-sectional data published from 2004 to 2018, the number of ALS patients showed a marked trend towards a considerably higher number of included patients in the more recent studies (Spearman Rank order correlation between number of included subjects per year and the year of publication, *ρ* = 0.87, *p* < 0.0001), whereas the total number of published studies over years of publication remained constant (*ρ* = 0.41, *p* = 0.12, Spearman Rank order correlation) (Supplementary Figure 1). Ten longitudinal studies were published between 2007 and 2018.

### Meta-analysis on cross-sectional data

Cross-sectional data from 57 studies (Table 1) included a total of *n* = 2064 ALS patients (58% males) and *n* = 1688 controls (52% males). Two of the 57 studies reported a very few cases with a family history of ALS (8 cases) for whom no detailed genetic testing was reported. The grand mean of patients age was 59 ± 11 years (range 46–65 years), the grand mean disease duration was 24 ± 20 months (range 3–62), the grand mean ALS functional rating scale (ALS-FRS-R)^12^ score was 37 ± 7 (range 27–41), and the grand mean disease progression rate computed as (48-ALS-FRS-R)/month of disease duration)^13^ was 0.5 ± 0.3 (range 0.2–1.4). Site-of-onset was reported in 35 cross-sectional studies indicating 27% patients (*n* = 222) with bulbar onset and 73% (*n* = 590) with spinal onset. However, almost all studies made no allowance for differentiating between different site-of-onset.

**Table 1:** Published cross-sectional studies included in the meta-analysis.

Study	Total number ALS patients (males/females)	Total number controls (males/females)	Mean age ALS-patients/years	Disease duration/months	ALS-FRS-R	Site of onset bulbar/spinal
Abe *et al*., 2004	7 (3/4)	11 (5/6)	57 ± 6	20 ± 13	n.a.	n.a.
Agosta *et al*., 2010	24 (13/11)	18 (11/7)	55 ± 13	34 ± 19	29 ± 4	6/18
Agosta *et al*., 2011	26 (15/11)	15 (8/7)	63	20	36	6/20
Agosta *et al*., 2016	48 (21/27)	51 (21/30)	59 ± 8	51 ± 51	37 ± 6	n.a.
Alruwaili *et al*., 2018	30 (19/11)	19 (8/11)	62 ± 11	22 ± 22	39 ± 5	4/26
Bartels *et al*., 2008	13 (5/8)	13 (5/8)	60 ± 7	20 ± 10	29 ± 6	6/7
Bastin *et al*., 2013	30 (17/13)	30 (16/14)	58 ± 11	24 ± 18	39 ± 7	n.a.
Ben Bashat *et al*., 2011	24 (n.a.)	22 (n.a.)	46 ± 9	25 ± 15	34 ± 9	n.a.
Blain *et al*., 2011	20 (14/6)	20 (12/8)	56 ± 11	28 ± 18	38 ± 6	4/16
Borsodi *et al*., 2017	27 (17/10)	35 (23/12)	58 ± 12	16 ± 13	39 ± 9	16/11
Buchanan *et al*., 2015	30 (17/13)	30 (16/14)	58 ± 11	24 ± 18	39 ± 7	10/20
Carrara *et al*., 2012	43 (26/17)	43 (26/17)	63 ± 11	14 ± 8	41 ± 5	n.a.
Christidi *et al*., 2014	21 (10/11)	11 (4/7)	62 ± 10	18 ± 18	40 ± 3	6/15
Christidi *et al*., 2017	42 (24/18)	25 (13/12)	62 ± 10	15 ± 13	40 ± 6	10/32
Corbo *et al*., 2014	19 (9/10)	19 (9/10)	62 ± 11	41 ± 31	34 ± 9	n.a.
Crespi *et al*., 2014	22 (15/7)	55 (32/23)	60 ± 10	23 ± 21	40 ± 9	4/18
Crespi *et al*., 2016	13 (10/3)	14 (9/5)	59 ± 11	25 ± 22	39 ± 6	3/10
de Albuquerque *et al*., 2017	53 (34/19)	57 (38/19)	56	19	35	9/43
Douaud *et al*., 2011	25 (18/7)	15 (9/6)	59 ± 12	44 ± 36	34 ± 4	6/19
Filippini *et al*., 2010	24 (17/7)	24 (16/8)	58 ± 12	49 ± 38	33 ± 4	3/21
Foerster *et al*., 2014	29 (17/12)	30 (20/10)	60 ± 10	29 ± 15	34 ± 8	7/22
Furtula *et al*., 2013	14 (9/5)	30 (12/18)	65 ± 7	33	39	3/11
van der Graaff *et al*., 2011	24 (15/9)	12 (7/5)	57	10 ± 3	40 ± 5	5/11
Hong *et al*., 2004	16 (9/7)	11 (5/6)	51 ± 12	11 ± 6	n.a.	n.a.
Iwata *et al*., 2011	18 (9/9)	19 (11/8)	53 ± 10	30 ± 18	36 ± 7	n.a.
Kassubek *et al*., 2018	387 (221/166)	144 (74/70)	60 ± 12	20 ± 18	39 ± 7	112/275
Keil *et al*., 2012	24 (12/12)	24 (n.a)	62 ± 11	26 ± 28	36 ± 9	9/15
Kim *et al*., 2014	14 (8/6)	16 (3/13)	54 ± 13	17 ± 7	37 ± 6	5/9
Kwan *et al*., 2013	23 (12/11)	19 (11/8)	56 ± 8	14 ± 7	34 ± 7	n.a.
Langkammer *et al*., 2010	15 (10/5)	15 (10/5)	60 ± 9	26 ± 16	39 ± 6	6/9
Li *et al*., 2009	10 (6/4)	10 (6/4)	46	21	38	n.a.
Lulé *et al*., 2010	14 (8/6)	18 (7/11)	55 ± 13	28 ± 27	33 ± 8	0/14
Metwalli *et al*., 2010	12 (10/2)	19 (11/8)	56 ± 11	26 ± 15	41 ± 5	41 ± 5
Mitsumoto *et al*., 2007	43 (31/12)	29 (10/19)	53 ± 11	30 ± 40	36 ± 8	n.a.
Müller *et al*., 2011	19 (9/10)	19 (9/10)	56 ± 12	n.a.	34 ± 9	n.a.
Müller *et al*., 2016	253 (140/113)	189 (96/93)	60	n.a.	37 ± 7	n.a.
Pettit *et al*., 2013	30 (17/13)	30 (17/13)	58	20	39	10/20
Rajagopalan and Pioro, 2017	21 (14/7)	14 (n.a.)	52 ± 11	9 ± 9	35 ± 8	n.a.
Rajagopalan *et al*., 2013	23 (13/10)	12 (8/4)	59 ± 13	29 ± 27	37 ± 9	n.a.
Rosskopf *et al*., 2015	140 (92/48)	139 (62/77)	62	28 ± 31	35 ± 8	n.a.
Sach *et al*., 2004	15 (10/5)	12 (n.a.)	52 ± 12	12 ± 6	n.a.	6/9
Sarica *et al*., 2014	14 (7/7)	14 (7/7)	62	29	27	9/6
Sarica *et al*., 2017	24 (11/13)	24 (9/15)	61 ± 9	19 ± 19	28 ± 7	7/17
Sarro *et al*., 2011	16 (8/8)	15 (7/8)	61 ± 10	29 ± 19	33 ± 7	1/15
Sato *et al*., 2010	15 (n.a.)	9 (n.a.)	60 ± 10	n.a.	n.a.	n.a.
Schuster *et al*., 2016	62 (36/26)	55 (29/26)	61 ± 9	30 ± 18	36 ± 7	26/36
Senda *et al*., 2011	17 (8/9)	17 (8/9)	61 ± 8	38 ± 19	37 ± 6	5/12
Sheelakumari *et al*., 2016	17 (7/10)	15 (8/7)	54 ± 14	11 ± 8	n.a.	n.a.
Spinelli *et al*., 2016	55 (31/24)	56 (25/31)	62	20 ± 17	36 ± 8	n.a.
Stagg *et al*., 2013	13 (7/6)	14 (n.a.)	62 ± 13	62 ± 55	32 ± 7	2/11
Stanton *et al*., 2009	20 (13/7)	21 (12/9)	58 ± 10	28 ± 18	39	3/17
Valsasina *et al*., 2007	28 (16/12)	20 (11)	55	26	27	n.a.
Vora *et al*., 2016	21 (14/7)	13 (8/5)	51 ± 15	n.a.	n.a.	n.a.
Wong *et al*., 2007	14 (5/9)	15 (8/7)	53 ± 14	22 ± 12	30 ± 6	2/12
Zhang *et al*., 2007	8 (6/2)	8 (6/2)	60 ± 11	n.a.	n.a.	n.a.
Zhang *et al*., 2011	17 (10/7)	19 (10/9)	57 ± 10	21 ± 10	35 ± 7	3/14
Zhang *et al*., 2017	38 (25/13)	35 (21/14)	49 ± 9	21 ± 18	31 ± 7	7/31

Listed are the total number of ALS patients and healthy controls, the mean age of ALS patients, the disease duration (months from disease defining symptoms onset), the revised ALS functional rating scale (ALSFRS-R), and the site of onset (bulbar/spinal) for each individual study. Age, disease duration, and ALSFRS-R are given as median or mean ± standard deviation, respectively. n.a – not available.

**Figure 2:**
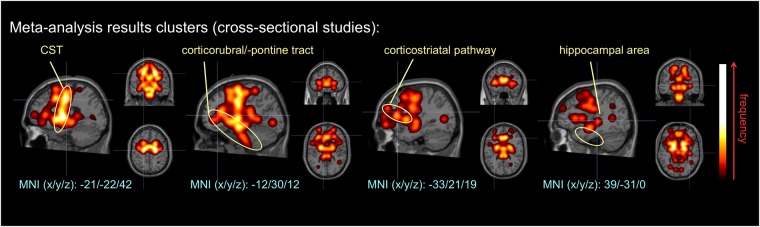
Clustered data from meta-analysis of 57 studies with cross-sectional data. Microstructural alterations were observed along the CST, along the corticorubral and the corticopontine tracts, the corticostriatal pathway, and in the perforant path.

The 57 individual DTI studies showed a total of 621 alteration locations resulting from significant FA differences between ALS patients and controls. The overlay of all 621 alteration locations indicated microstructural alterations along the CST (related to ALS stage 1), frontal and midbrain regions along the corticorubral and the corticopontine tracts (related to ALS stage 2), along the corticostriatal pathway (related to ALS stage 3), and in hippocampal regions (related to ALS stage 4). The involved brain regions, as revealed by the final statistical meta-analysis data, are summarized in Fig. 2. A fixed-effects meta-analysis was conducted to combine the results of the included studies.

### Meta-analysis on longitudinal data

Longitudinal data from 10 studies (Table 2) included a total *n* = 266 ALS patients (64% males) and *n* = 222 controls (56% males) who underwent baseline investigation and follow-up after 8 months (grand average). No data on family history was reported. For baseline data, the grand mean of patients age of onset was 57 ± 6 years (range 41–62 years), the grand mean disease duration was 28 ± 21 months (range 15–44 months), the grand mean ALS functional rating scale (ALS-FRS-R)^12^ score was 38 ± 8 (range 31–41), and the grand mean disease progression rate^13^ was 0.5 ± 0.1 (range 0.2–0.6). Site-of-onset when reported indicated 71% with spinal onset and 29% patients with bulbar onset (Table 2).

**Table 2:** Published longitudinal studies included in the meta-analysis. Listed are the total number of ALS patients and healthy controls, the mean age of ALS patients, the disease duration (months from disease defining symptoms onset), the revised ALS functional rating scale (ALSFRS-R), the site of onset (bulbar/spinal), and the time interval (months) between baseline and follow-up assessment for each individual study. Age, disease duration, and ALSFRS-R are given as median or mean ± standard deviation, respectively. Demographical and clinical data refer to baseline assessment. n.a – not available. ^+^values provided for follow-up assessment.

Study	Total number ALS patients (males/females)	Total number controls (males/females)	Mean age ALS-patients/years	Disease duration/months	ALS-FRS-R	Site of onset bulbar/spinal	Time difference/month
Cardenas-Blanco *et al*., 2016	34 (12/22)	29 (13/16)	57 ± 10	24 ± 21	40 ± 4	n.a.	8
de Albuquerque *et al*., 2017	24 (15/9)	27 (11/16)	41	31	31	2/12	8
Kassubek *et al*., 2018	67 (43/24)	31 (20/11)	62 ± 10	28 ± 18	40 ± 5	18/49	9
Keil *et al*., 2012	24 (12/12)	24 (n.a.)	62 ± 11	26 ± 28	36 ± 9	9/15	6
Kwan *et al*., 2012	9 (5/4)	19 (11/8)	57 ± 12	44 ± 35	40 ± 6	n.a.	15
Mitsumoto *et al*., 2007	43 (31/12)	29 (10/19)	53 ± 11	30 ± 40	36 ± 8	n.a.	n.a.
Senda *et al*., 2011	17 (8/9)	17 (8/9)	61 ± 8	38 ± 19	37 ± 6	5/12	6
Steinbach *et al*., 2015	15 (14/1)	15 (14/1)	62 ± 12	15 ± 11	41 ± 4	5/10	3
van der Graaff *et al*., 2011	16 (n.a.)	12 (7/5)	57 ± 10	n.a.	n.a.	9/7	6
Zhang *et al*., 2011	17 (10/7)	19 (10/9)	n.a.	21 ± 10	35 ± 7^+^	3/14	8

**Figure 3:**
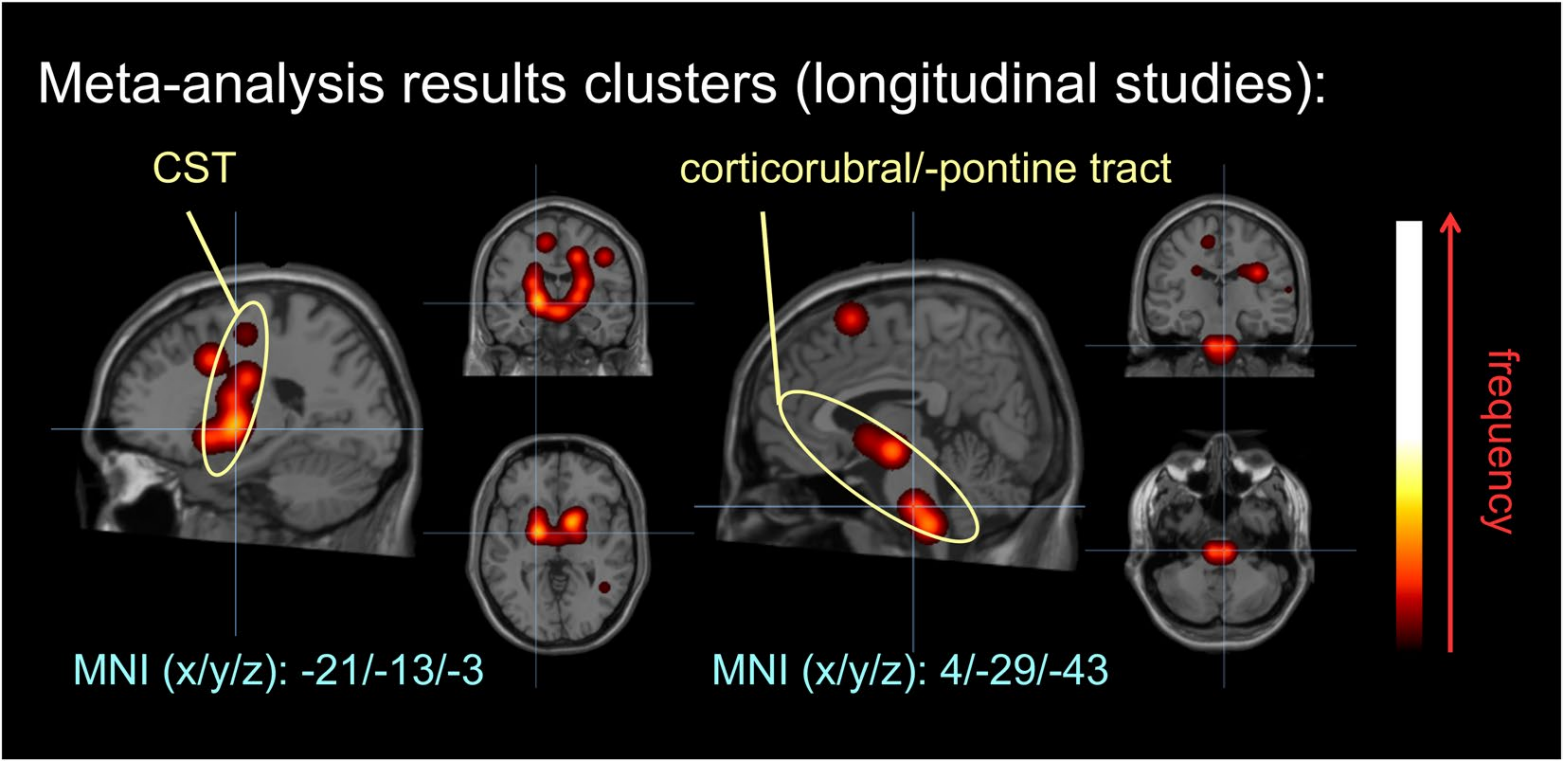
Clustered data from meta-analysis of 10 studies with longitudinal data. Microstructural alterations were observed along the CST and along the corticorubral and the corticopontine tracts over time.

The 10 individual DTI studies revealed a total of 38 clusters indicating significant differences in ALS over time. Using a fixed-effect model as for the cross-sectional data analyses, the overlay of 38 alteration locations indicated changes most prominent in the CST (related to stage 1) and corticorubral/-pontine tract (related to stage 2) over time (Fig. 3). Changes in stage 1 and stage 2-related tracts are in full support with both cross-sectional meta data and the proposed staging model, indicating pathology progression in the stage 1-related CST and the progressive impairment in regions associated with stage 2.

### Similar results of meta-analysis and monocentric large-scale study in voxelwise statistics

**Figure 4:**
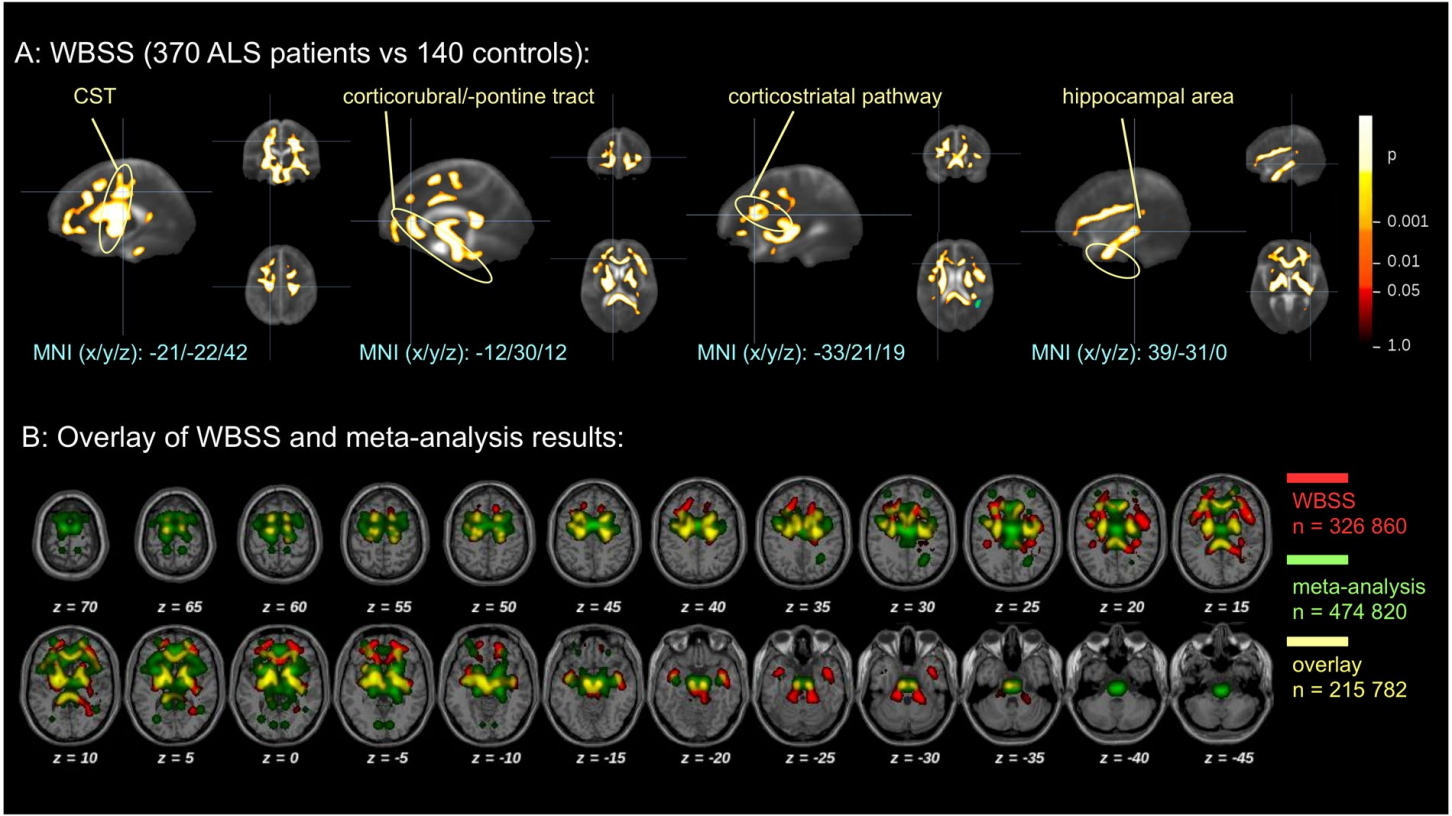
(**A**) Clustered data from a monocentric whole brain-based spatial statistics of FA maps of 370 ALS patients vs 140 controls. Microstructural alterations were observed along the CST, along the corticorubral and the corticopontine tracts, the corticostriatal pathway, and in hippocampal regions. **(B)** Overlay of WBSS and meta-analysis results.

Whole brain-based spatial statistics (WBSS) of a large monocentric data sample of FA maps (*n* = 370 ALS patients vs. *n* = 140 controls) demonstrated similar results compared to the meta-analysis (Fig. 4A,B). As in the cross-sectional meta-analysis results, microstructural FA alterations were detected along the CST, frontal and midbrain regions along the corticorubral and the corticopontine tracts, along the corticostriatal pathway, and in hippocampal regions. An overlay analysis of results of the meta-analysis (*n* = 474,820 voxels) and the large mono-centric study (*n* = 326,860 voxels) revealed an overlap of *n* = 215,782 voxels from both analyses.

### The association between structural alteration and neuropathological disease stages

**Figure 5:**
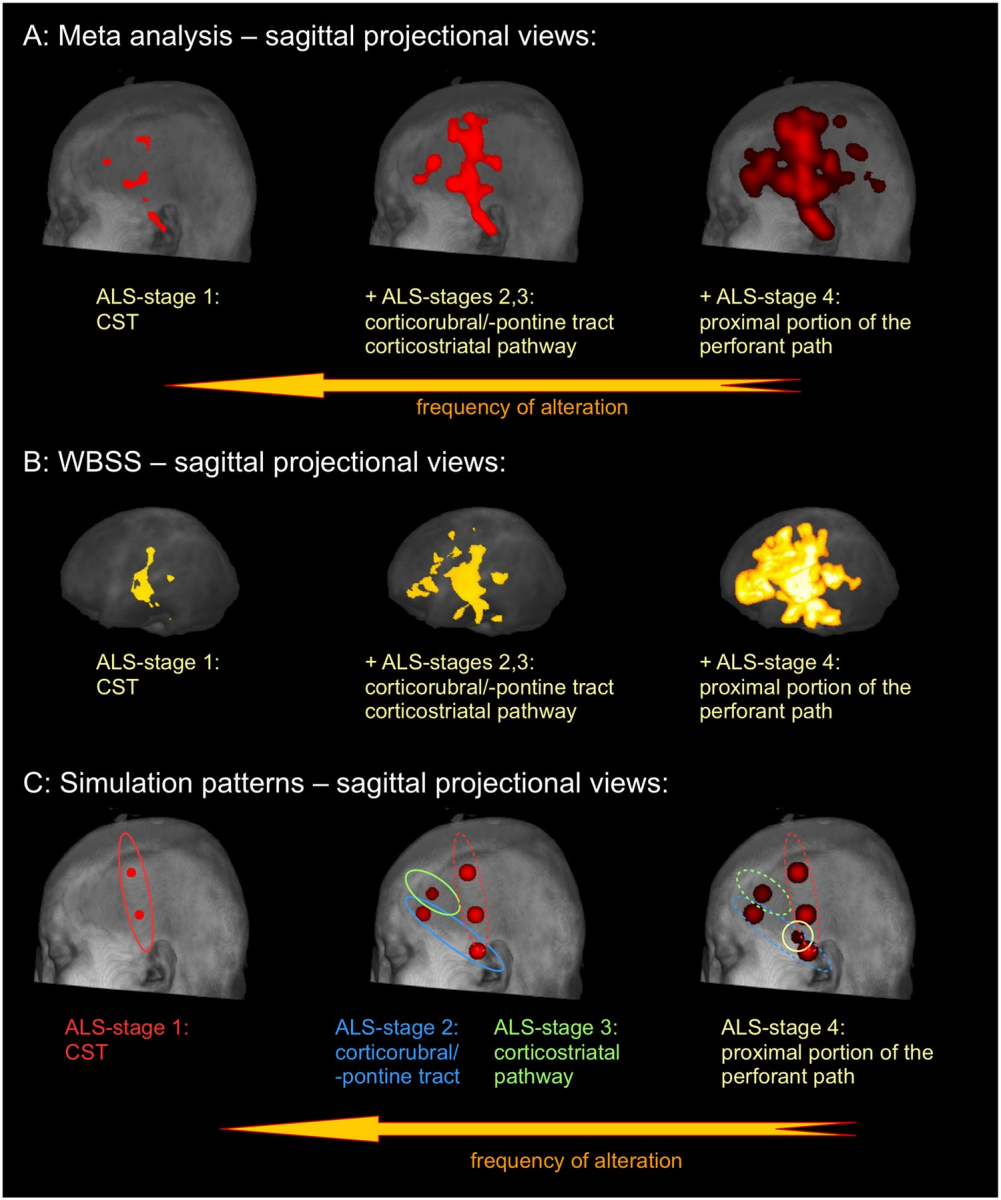
Results at different statistical power thresholds revealed a pattern of sequential involvement of ALS-specific white matter regions. Specific involvement of the CST at the highest threshold (ALS stage 1), followed by additional involvement of corticorubral/-pontine tract together with corticostriatal pathways at a lower frequency of alteration threshold (ALS stages 2 and 3), and. finally, revealing also hippocampal involvement for the lowest threshold corresponding to the proximal portion of the perforant path (ALS stage 4). Hot colors encode the frequency of alteration. (**A**) Results of the meta-analysis. (**B**) Results clusters of the WBSS of FA maps. (**C**) Simulation pattern of ALS-associated spread of TDP-43 pathology.

To address the pattern of sequential involvement of ALS-associated tract systems, the frequency of alterations was analyzed. This analysis resembled the pattern of sequential involvement of ALS-specific tracts (Fig. 5A). Alteration frequencies were calculated in percent of the most frequent alteration location (which was defined as 100%). In particular, the meta-data demonstrated specific involvement of the CST at the highest alteration frequency level (thresholded at 75% of most frequent alteration location). Additional involvement of the corticorubral/-pontine tract together with the corticostriatal pathway was observed at a threshold level of 50% (of most frequent alteration location) and, finally, the hippocampal involvement (the proximal portion of the perforant path) was demonstrated at a threshold level of 25% (of most frequent alteration location). A similar pattern could be observed in the WBSS analysis from the mono-centric study. The highest significances were found along the CST (ALS stage 1) (*p* < 0.00001, FDR corrected), followed by significances along the corticopontine/-rubral tract and the corticostriatal pathway (ALS stages 2 and 3) (*p* < 0.001, FDR corrected), significant alterations (*p* < 0.01, FDR corrected) were finally found also in hippocampal regions adjacent to the proximal portion of the perforant path (ALS stage 4).

Finally, the simulation pattern of sequential involvement of brain regions corresponding to disease stages showed a similar significance alteration pattern: first, involvement of the CST (thresholded at 75% of most frequent alteration location), followed by additional involvement of the corticorubral/-pontine tract together with the corticostriatal pathway (thresholded at 50% and 25%, respectively, of most frequent alteration location), and, finally, also hippocampal involvement (thresholded at 0% of most frequent alteration location). Thus, the simulation scenario demonstrates that the simulation of sequential involvement is a possible model for the disease alteration pattern revealed by the meta-analysis and WBSS analysis. However, it must be emphasized that the simulation pattern of the present study is not the only scenario that could lead to a pattern resembling the meta-analysis result pattern.

## Discussion

By using a meta-analysis approach, DTI data from 57 cross-sectional studies with *n* = 7 up to *n* = 387 ALS patients were pooled to investigate microstructural differences in ALS patients compared to controls. This study specifically aimed at investigating if the alteration pattern from DTI metadata resembled the sequential spread of pTDP-43 pathology in ALS according to *post-mortem* data^3^ which had previously been transferred into a DTI-based approach for the *in vivo* classification to ALS stages^7,8,14^. Our working hypothesis that this DTI correlate of the neuropathological propagation pattern can be demonstrated in the meta-data similar to previous large monocenter^8^ and pooled multicenter data^14^ was indeed verified by our/the observation of microstructural alterations along the CST, the corticorubral and the corticopontine tracts, the corticostriatal pathway, and in the perforant path. By varying the significance threshold for group differences, this disease-specific pathological pattern of regional involvement was consistent with histopathological findings. Meta data analyses on 10 longitudinal studies strongly support a propagation model by revealing alterations in regions associated with stage 1 and 2 over time. The (cross-sectional) analysis of a large-scale monocentric data sample showed a high overlap with these results. Simulations modelled the overall results as revealed by *post-mortem* data^3^, thus indicating a potential scenario for disease propagation and progression.

Our approach adds to the results of a recent meta-analysis of eight previous studies^15^. The current study included a comparatively large number of 59 studies, resulting in a cluster distribution in regions known to become sequentially involved during ALS in accordance with the proposed pattern of pTDP-43 pathology. In particular, the statistical power of the reported brain regions from the cross-sectional studies included presented a marked gradient with the highest frequency in brain regions associated with ALS stage 1 towards the relatively lowest frequencies associated with ALS stage 4. These findings were supported by longitudinal meta data that indicated alterations in the CST (according to stage 1) and corticorubral/-pontine tracts (according to state 2) over time, i.e., areas associated with stage 2 become involved while pathology worsened in regions associated with state 1. Areas associated with stage 3 and 4 failed to reach significance since these advanced stages were reached only in a subgroup of the patients. The most obvious explanation for this situation derives from the nature of ALS patient recruitment for MRI studies: It becomes increasingly challenging to run MRI scans in individuals with severe physical disability at later disease stages. We aimed to integrate as much information from as many studies as possible so that we could minimize a bias from studies with large sample sizes.

Three aspects should be emphasized within this context: First, the vast majority of reported results originate from cross-sectional data. Second, the cluster results revealed a conglomerate of microstructural alterations that might originate from ALS patients with a homogeneous distribution over all ALS stages. Third, we assumed that DTI-based results obtained in the early 2000s could be identically weighted compared to results created obtained recently.

Our study is not without limitations. A potential drawback of the approach is the limited number of longitudinal studies available and that meta studies *per se* provide results at the group level. However, a recent longitudinal DTI study strongly supports a sequential disease propagation of pTDP-43 pathology *in vivo* by providing data at the individual level^8^. The data available from the individual studies did not allow for the characterization of clinical and demographical features at each stage of the disease. The simulation scenario showed a homogeneous distribution of ALS patients over all ALS stages, thereby demonstrating a dependency of the statistical power on these stages. In addition, the meta-analysis results showed a dependency of the statistical power on the four ALS stages. However, we should point out that the simulation pattern of our study is not the only scenario that would lead to a pattern that coincides with the meta-analysis alteration pattern, and that the proposed four-stage pattern for sporadic (TDP-43) ALS is not applicable to *SOD1* and *FUS* ALS. Other scenarios are also possible, but the detailed scenario of the present study can serve as a possible model. Generalized values were used (10 mm seed size, identical significances) for the analysis of the pattern of FA alterations, so that within-study clusters were not differentiated. The potential functional consequences of the different disease stages remain open when behavioral information beyond the routinely reported physical impairment are incorporated. Therefore, future studies should also analyze the dependency of identified ALS disease stages and an association with more detailed clinical and neuropsychological scores. Further longitudinal studies are now warranted to assess whether the proposed distribution of TDP-43 pathology can be further replicated from longitudinal data. Our meta analysis calls for increasing the number of longitudinal studies, possibly conducted in patients at the earliest phase of the disease, so as to track the tract involvement at the individual level during the course of ALS.

In conclusion, we present new *in vivo* neuroimaging evidence from a systematic meta-analysis for the proposed neuropathological staging concept of ALS. This evidence supports the potential of DTI to map the *in vivo* pathoanatomy of ALS non-invasively in accordance with *post-mortem* neuroanatomical studies. As discussed previously^14^, DTI metrics might serve as potential read-outs and/or potential biomarkers of ALS and its progression for future clinical trials. As such, advanced neuroimaging might, at the mechanistic level, guide us in the understanding of the underlying pathomechanisms in ALS.

## Methods

### Meta-analysis: Search strategy and study selection

A systematic literature search using the search engines PubMed and Scopus for databases from 1990 to July 2018 was carried out using the keywords “amyotrophic lateral sclerosis” OR “ALS” OR “motor neuron disease” AND “diffusion tensor” OR “DTI.” In addition, the reference lists of the relevant studies and Google Scholar® were searched for additional studies. The selection criteria were:

- human studies
- whole-brain analyses
- papers published in English in peer-reviewed journals
- ALS patients met the diagnostic criteria according common diagnostic guidelines^16,17^
- thresholds for significance corrected for multiple comparisons
- statistical comparison of whole-brain based FA values between manifest ALS patients compared to controls or longitudinal studies in ALS patients
- clusters indicating statistically significant FA values differences are provided in a common stereotaxic space, i.e., in Talairach coordinates^18^ or Montreal Neurological Institute (MNI) coordinates^19^ or given as precise anatomical location that could be quantitatively related to an axonal fiber tract.

### Meta-data analysis in stereotaxic space

The publications used in this analysis were screened for FA differences between ALS patients and controls. All data from brain regions (cluster size, stereotaxic brain coordinates, significance level of FA differences) were analysed. The *Tensor Imaging and Fiber Tracking* (*TIFT*) software package was used for data analysis^20,21^.

All extracted coordinates were provided as MNI coordinates for statistical analysis. To that end, coordinates given in Talairach space were transformed into the MNI stereotaxic space according to a standardized transformation procedure^22,23^. If coordinates of FA differences were not given as a center of mass or peak likelihood, brain MNI coordinates were estimated based on the precise description of the anatomical locations by an online MNI brain atlas (http://sprout022.sprout.yale.edu/mni2tal/mni2tal.html, February 1^st^, 2018). The meta-analysis framework of this study was performed by weighting the individual study results by the binary logarithm of the number of included subjects (*w*_*N*_ = lb(*N*)), thereby reducing the effect that recent studies with a larger number of subjects (*N* > 200) might bias the results. This technique allowed us to correct for different effect sizes of the respective clusters from the individual studies. A fixed-effects model was used to combine the results of 57 cross-sectional and 10 longitudinal studies included in this meta-analysis.

Of the 57 studies with cross-sectional data, a total of 621 coordinates indicating significant FA differences between ALS patients and controls were transformed into a 1 mm isogrid (default *TIFT* resolution^20^) by using a seed sphere (*d* = 10 mm) placed with its center at the respective 3-D MNI coordinates. The same procedure was applied to the 10 studies with longitudinal data revealing a total of 38 coordinates indicating significant FA changes in ALS patients over time.

The meta-analysis framework of our study was performed by weighting the individual study results by the binary logarithm of the number of included subjects (*w*_*N*_ = lb(*N*)), thereby reducing the effect that recent studies with a large number of subjects (*N* > 200) might bias the results. This technique permitted us to correct for different effect sizes of the respective clusters from the individual studies.

The grand average map was finally computed by using spatial smoothing with an isotropic Gaussian kernel^24^ of full-width-at-half-maximum (FWHM) size of 12 mm to optimally balance between sensitivity and specificity, as it is commonly done in the whole-brain-based spatial statistics (WBSS) framework^14,25^. It is of note that using a Gaussian kernel also provides an indicator of proximity of the reported x-y-z foci and smooths intra-dataset and between-dataset variability^26^. The statistical outcomes were further corrected for multiple comparisons using the false-discovery-rate (FDR) algorithm at *p* < 0.05^27^. Further reduction of the alpha error was performed by a spatial correction algorithm that eliminated isolated voxels or small isolated groups of voxels in the size range of the smoothing kernel leading to a cluster size threshold of 256 voxels.

White matter alterations in relation to ALS-associated TDP-43 pathology propagation^3^ were investigated by variation of the significance threshold of the statistical across discrete steps, with stage 1 referring to the regions implicated in ALS cases with the least extensive patterns of microstructural alterations in FA: in stage 1 along the CST, in stage 2 in the CST and in frontal and midbrain regions along the corticorubral and the corticopontine tracts, in stage 3 in frontal regions along the corticostriatal pathway, and in stage 4 in all previously mentioned brain structures as well as in the proximal portion of the perforant path^7,8^. To this end, altered white matter structures with the highest statistical power were attributed to stage 1 and those with least statistical power that will be attributed to stage 4.

Finally, the results were subjected to a simulation analysis of FA alterations. An involvment pattern was simulated according to stages: By variation of the statistical power threshold, the remaining pattern could be assigned to the ALS propagation pattern (as the basis of the simulation), i.e., microstructural alterations along the CST (stage 1), in frontal and midbrain regions along the corticorubral and corticopontine tracts (stage 2), the corticostriatal pathway (stage 3), and perforant path (stage 4).

### Cross-sectional whole brain-based spatial statistics of ***in vivo*** DTI data

The DTI data set for WBSS was selected from the monocentric data base of the Department of Neurology at the University of Ulm, Ulm, Germany. These DTI data have been analyzed previously: For details of the patient characterization and data analysis refer to Kassubek *et al*.^8^. The data-set consisted of 510 cross-sectional DTI data from ALS patients (*N* = 370) and controls (*N* = 140) acquired at 1.5 T (Magnetom Symphony, Siemens Medical, Erlangen, Germany) or at 3.0 T (Allegra, Siemens Medical, Erlangen, Germany).

### Simulation of ALS pathology spreading

According to the model of sequential transaxonal disease spread in ALS and its *in vivo* correlates in MRI^5,7,8^, a computational model according to previous studies^7,14^ was used, which had shown an involvement of white matter tracts during the course of the disease that allows for staging categorization. ALS patients in stage 1 show microstructural alterations along the CST, those in stage 2 show microstructural alterations in the CST as well as in frontal and midbrain regions along the corticorubral and the corticopontine tracts. ALS patients in stage 3 additionally show microstructural alterations in frontal regions along the corticostriatal pathway, and, in stage 4, patients show alterations in all aforementioned brain structures and additionally in the proximal portion of the perforant path^7,8^.

If an ALS-patient sample is assumed that consists of patients homogeneously distributed over all ALS stages, i.e., 25% of the ALS patients are in each of the four ALS stages, a simulation could be performed that showed microstructural alterations along the CST in 25% of the subjects (ALS stage 1) and microstructural alterations along the CST as well as in frontal and midbrain regions along the corticorubral and the corticopontine tracts in 25% of the subjects (ALS stage 2). The same applies to the following stages: 25% of the subjects manifested microstructural alterations along the CST, in frontal and midbrain regions along the corticorubral and the corticopontine tracts, and, additionally, along the corticostriatal pathway (ALS stage 3); finally, 25% of the subjects displayed microstructural alterations in all of the previous ALS-associated brain regions plus the proximal portion of the perforant path (ALS stage 4). This involvement was simulated in the identical approach as the meta-data using a seed sphere (*d* = 10 mm) placed with its center at the respective 3-D MNI coordinates followed by a spatial smoothing with an isotropic Gaussian kernel with FWHM of 8 mm^28^.

## Electronic supplementary material

SupplementaryFigure
