# Supplementary material for: Corticoefferent pathology distribution in amyotrophic lateral sclerosis: in vivo evidence from a meta-analysis of diffusion tensor imaging data
Source: Sci Rep. 2018 Oct 18;8:15389. doi: 10.1038/s41598-018-33830-z (PMC6194130; doi:10.1038/s41598-018-33830-z)
Supplement: Supplementary file 1 — SupplementaryFigure [file 41598_2018_33830_MOESM1_ESM.docx]

**Corticoefferent pathology distribution in amyotrophic lateral sclerosis: *in vivo* evidence from a meta-analysis of diffusion tensor imaging data**

**Martin Gorges^1^, Kelly Del Tredici^1,2^, Jens Dreyhaupt^3^, Heiko Braak^1,2^, Albert C. Ludolph^1^, Hans-Peter Müller^1,+^, Jan Kassubek^1,+,^***

^1^Department of Neurology, University of Ulm, Germany

^2^Clinical Neuroanatomy, Department of Neurology, University of Ulm, Ulm, Germany,

^3^Institute of Epidemiology and Medical Biometry, University of Ulm, Germany

*jan.kassubek@uni-ulm.de

^+^these authors shared senior authorship.


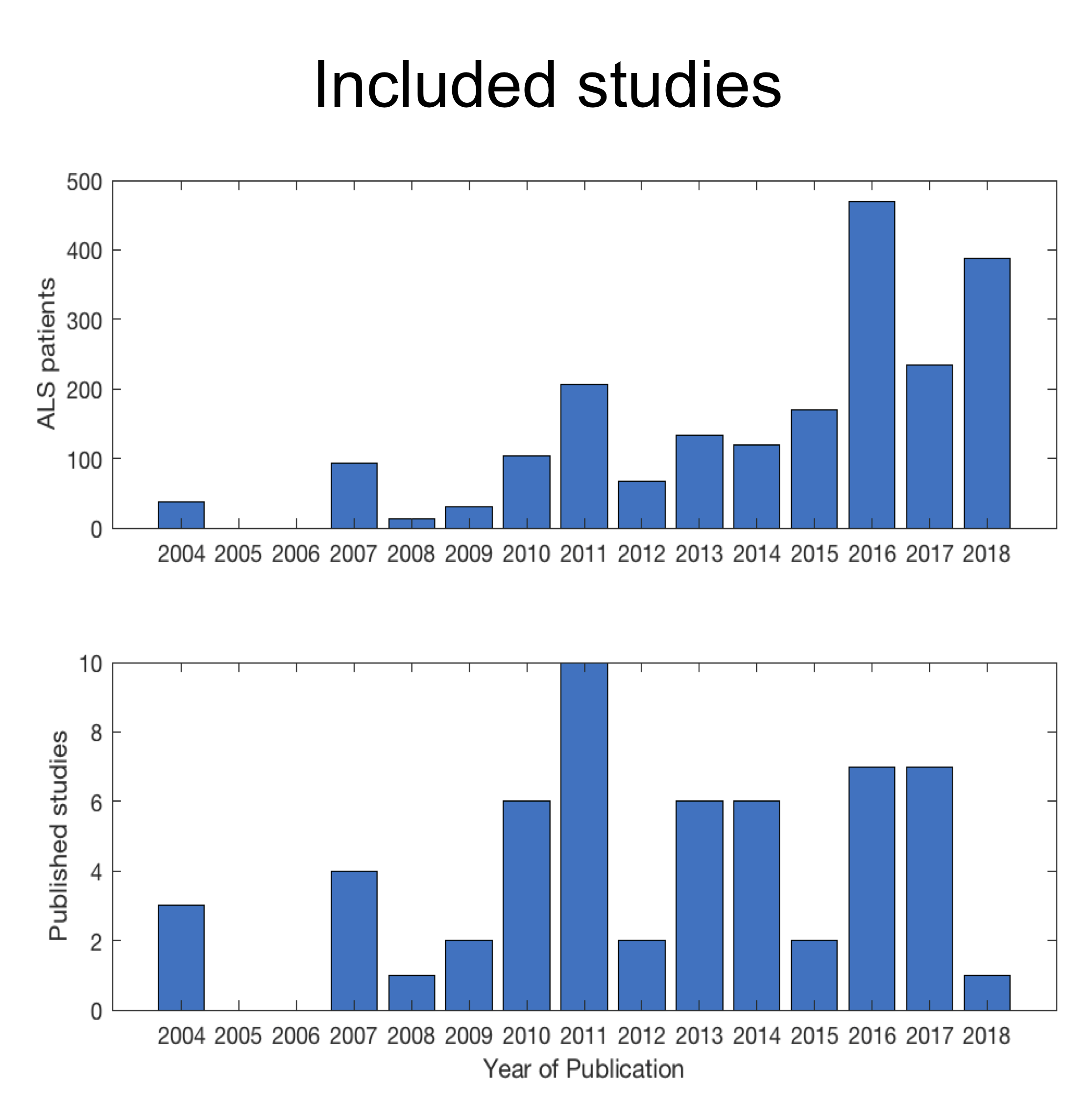


**Supplementary Figure 1.** Statistically significant correlation (*ρ*=0.87, *p*<0.0001***, Spearman Rank order correlation) between the number of ALS patients included in the 57 studies with cross-sectional data and the year of publication indicating a marked trend towards a considerably higher number of patients included in the more recent studies (upper row), whereas the number of published studies did not significantly increase (*ρ*=0.41, *p*=0.12, Spearman Rank order correlation) over the years of publication (lower row).
